# Ring chromosome 13 syndrome characterized by high resolution array based comparative genomic hybridization in patient with 47, XYY syndrome: a case report

**DOI:** 10.1186/1752-1947-5-99

**Published:** 2011-03-11

**Authors:** Can Liao, Fang Fu, Liang Zhang

**Affiliations:** 1 Department of clinical genetics department, Guangzhou Women and Children' Medical Center, Guangzhou Medical College, Guangzhou, Guangdong 510623, PR China; 2 National Engineering Research Center for Beijing Biochip Technology, Beijing 102206, PR China

## Abstract

**Introduction:**

The co-occurrence of ring chromosome 13 syndrome and 47, XYY syndrome in the same individual is rare. To the best of our knowledge, this is the first report of the co-existence of this kind of chromosome aberrations. At present, the deletion 13q syndrome is divided into three groups based on the deletion's location relative to chromosomal band 13q32. Group 1 (proximal to q32) and group 2 (including q32) have shown distinctive phenotypes including mental retardation and growth deficiency. Group 3 (q33-34 deletion) is defined by the presence of mental retardation but there is usually an absence of major malformations.

**Case presentation:**

We describe a 10-month-old Chinese Han boy presenting with severe mental retardation, profound congenital bilateral hearing loss with a terminal 13q33.2 deletion and multiple malformations. Routine chromosome analysis disclosed a *de novo *complex karyotype 47, XYY, r(13)(p11q34). Further investigation by high resolution array-based comparative genomic hybridization delineated an 8.5 Mb terminal deletion on the long arm of chromosome 13(13q33.2→q34).

**Conclusion:**

The co-occurrence of double syndromes in the same individual is rare and its clinical presentation is variable depending on the predominating abnormality or a combination of the effect of both. Hearing impairment is suggested as another new clinical feature to 13qter deletion. This case report will contribute to more accurate genetic counselling and provide further insight to the syndrome.

## Introduction

Ring chromosomes frequently arise following a breakage in the short and long arms of a chromosome with rejoining at the two ends to complete the ring. Ring chromosome 13 is relatively uncommon, with an estimated incidence of 1/58,000 in live births [1]. A ring chromosome formation of an acrocentric chromosome is often associated with increased severity of clinical symptoms compared to the deletion of the same segment but without ring formation [2]. The phenotype of patients with terminal deletion of chromosome 13 has a very large spectrum, which seems to depend on the location of the deleted segment. According to current classification, the syndrome is divided into three groups based on the deletion's location relative to chromosomal band 13q32. Group 1 (proximal to q32) and group 2 (including q32) have shown distinctive phenotypes including mental retardation and growth deficiency, whereas with group 3 it is suggested that breakpoints at 13q33 and 13q34 are frequently found in patients with severe mental retardation, microcephaly with true hypertelorism; frontal bossing erasing the nasal bridge, protruding upper incisors and large external ears with deep sulci. In addition, male patients frequently have genital malformations [3,4].

The 47, XYY syndrome is an aneuploidy of the sex chromosomes in which a human male receives an extra Y chromosome. This chromosomal anomaly occurs in approximately 1/1000 live male births but is more frequently found in the infertile population [5]. The phenotypic features of 47, XYY patients mostly remain normal except for a high risk of infertility and behavioral disorder [6]. However, the co-occurrence of such double syndromes in the same individual is rare. We report a case showing the co-existence of these numeric and structural chromosomal abnormalities which are characterized by a high resolution array based comparative genomic hybridization (aCGH).

## Case presentation

Our patient was a 10-month old Chinese Han ethnic boy. The pregnancy history was negative for significant complications or teratogenic exposures. He was born at full term by vaginal delivery with: birth weight 2.1 kg (3rd centile); length 47 cm (25th centile); head circumference 30 cm (3rd centile); and thorax circumference of 28 cm (3rd centile). The delivery procedure and neonatal period were not complicated. He was found to be unresponsive to sound. Physical examination at 10 months showed: head circumference 39.5 cm; weight 7.11 kg; mental retardation; microcephaly; microophthalmia; hypertelorism; large external ears; flat nasal bridge; and a broad gastroschisis with a short philtrum. A hearing test revealed bilateral severe sensorineural hearing loss. Brain magnetic resonance imaging (MRI) discovered corpus callosum hypoplasia and cerebral white matter abnormalities. Comprehensive neuropsychological testing indicated impaired functioning across most of the cognitive domains and delayed psychomotor development. Gross movement was equivalent to a two-months-old child, fine movements and cognition level were equal to three months and speech was equivalent to four months. Physical examination also revealed microorchidism and hypospadias.

## Cytogenetic analysis

Metaphase chromosome preparation from the proband was made from peripheral blood lymphocytse and chromosome analysis was performed on lymphocytes with standard methods at 450-500 band resolution. Chromosome analysis was also performed on both parents.

## Genome wide microarray analysis

Genomic DNA from whole blood was extracted and hybridized with the single-nucleotide polymorphism (SNP) 6.0 arrays using a Human Mapping SNP6.0 assay kit following the manufacturer's standard protocol. The data were analyzed by a comparison with those of the 96 hapmap Asian individuals used as control. Initial analysis and quality assessment of the array data were performed with Genotyping Console (Affymetrix, CA, USA). The median absolute pair-wise difference (MAPD) of each chip was used as a quality assessment of the array data. The median MAPD of this array was 0.26, which met the quality control criteria by Affymetrix. In order to avoid the loss of copy number variant (CNV) discovery, we merged the results from two software packages: Genotyping Console and Partek Genomics Suite, respectively. In order to minimize the potential false positive rate from signal-to-noise ratio, only CNVs that involved at least 10 consecutive probe sets were considered, thus, providing a median resolution of 30 kb.

## Results

**Figure 1:**
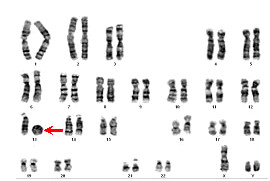
**Cytogenetic cartogram of the case: 47, XYY, r(13)(p11q34)**. The arrowhead demonstrates the ring chromosome 13.

Cytogenetic analysis showed 47 chromosomes including a small ring chromosome 13 and an extra Y in all the 30 analyzed cells. The karyotype of the propositus was 47, XYY, r(13)(p11q34) (see Figure 1). The karyotypes of both parents were normal.

**Figure 2:**
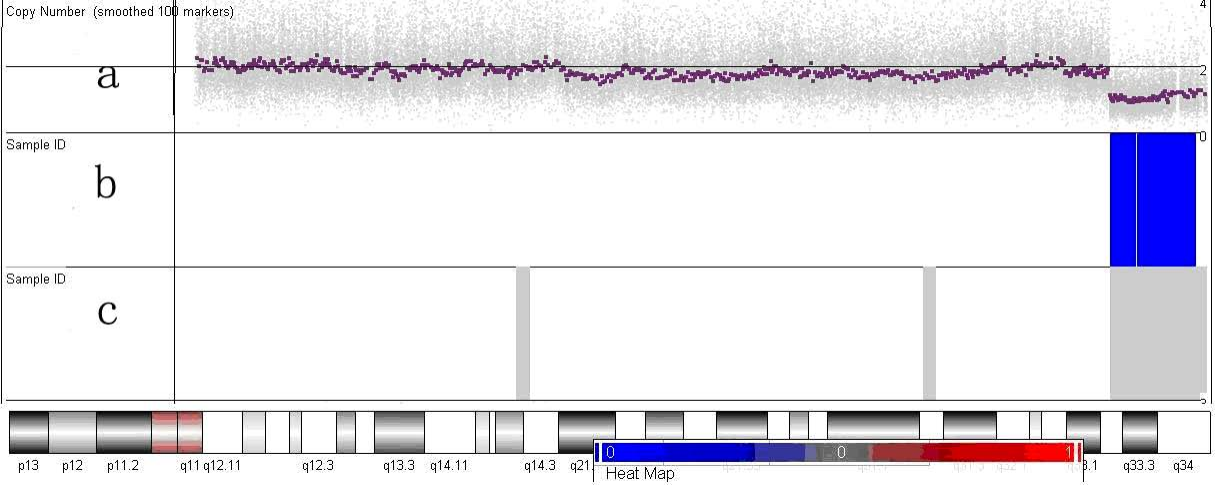
**Genome-wide array comparative genome hybridization result: 13q33**.2→qter deletion. There are three icons from top to down in total **(a-c)**. (a) A scatter plot of a copy number; a gray point shows the copy number calculated from a probe set and a red point represents the mean copy number calculated from consecutive 100 probe sets. The baseline in the middle indicates the normal copy number level. Upward deviation from the baseline indicates amplification and downward departure from the baseline represents deletion. (b) A schematic of copy number variant (CNV) segments identified based on the segmentation algorithm. The blue color represents deletion CNV. (c) A diagram of the loss of heterozygosity (LOH).

Genome wide SNP 6.0 array analysis revealed a 8.5 Mb terminal deletion from Affymetrix probe set SNP_A-8677827 to CN_636381, corresponding to the physical position from 104865782 bp to 113948665 bp, mapping to chromosome 13q33.2→qter (see Figure 2). There were no pathologic variants in the parents.

## Discussion

Ring chromosome 13 and 47, XYY are relatively rare syndromes in the human genome, respectively. To best the best of our knowledge, the co-existence of these two chromosomal abnormalities in same individual has not previously been reported and their genetic mechanisms are considered to be independent of each other. The formation of ring chromosome 13 may arise during meiosis or occur in the post-zygotic period [3,4], while 47, XYY karyotype without mosaic may be the result of the nondisjunction of the Y chromosome during meiosis. Although a routine cytogenetic analysis by G-banding can detect common trisomies and some apparently structural abnormalities, it takes time to culture cells and cannot detect deletions or duplications smaller than 4 Mb [7]. Recently, the advent of microarray-based comparative genomic hybridization (aCGH) technology permits the simultaneous rapid high resolution genome analysis and mapping of DNA sequences [8,9]. Since its development, aCGH has been applied mostly as a research tool in the field of the detection and identification of unbalanced chromosomal abnormalities in prenatal, postnatal and preimplantation diagnosis. In this study we use the high resolution aCGH to screen the whole genome and identified an 8.5 Mb terminal deletion of chromosome 13 to demonstrate the application of aCGH in clinical genetics diagnosis.

There are at least 120 reported cases of distal 13q deletions to date. According to the literature, the clinical manifestations of our patient are consistent with the major phenotypes of chromosome 13 deletion syndrome. Microorchidism and hypospadias in our patient plus those of reported male patients who have genital abnormalities, provides support for the hypothesis that 13q32.2-q34 region plays an important role in genital development and that gene EFNB2 locus in band q33.2 is a candidate gene for male genital malformations [10,11].

Severe brain malformation, including hypogenesis of corpus callosum plus cerebral white matter abnormalities in our patient and lumbosacral myelomeningocele and anencephaly in the reported cases [10-13], further confirm the hypothesis that a critical region existing between 13q33.2→qter is responsible for the development of nervous system in the 13q deletion syndrome [14]. However, one of the most notable features in our case is the congenial bilateral profound hearing loss and this clinical feature is uncommonly reported in either the terminal or interstitial deletion of chromosome 13.

We reviewed the previously reports and found that Kirchhoff *et al*. [15] described 13 patients with 13q deletion. Among them two (q32.3-q34 and q31.2-q34 deletion) were found to be having hearing impairment which was in accordance with our patient's deafness. We also noted that hearing impairment association with 13q deletion was reported in an animal model [16]. In their research they hybridized DNA sequences mapping to the human chromosome deletion of band 13q22→q32 to homologous sequences of mouse and, as result, the mouse-human hybridized model manifested partial hearing loss, mild mental retardation and minor dimorphic features. As, in the majority of cases with the 47, XYY syndrome, the phenotypic features are also not associated with hearing impairment, so deafness in our case was suggested to be another clinical features of the 13qter deletion. There are 69 genes within this deleted region and the haploinsufficiency of some gene(s) may contribute to this congenital malformation. More precisely, comparing to the breakpoint defined by Cowell and Mitchell (their mouse had only mild partial hearing loss), the possible causative gene/genes may locus in a more similar site to our patient's breakpoint. With regard to other patients with this rearrangement who do not have hearing impairment, we hypothesize that a chance association or low penetrance of the putative hearing gene/genes seems to be the likely explanation. Further gene content and more patient studies are needed in the future.

## Conclusion

Hearing impairment detected in our patient may be a clinical feature associated with the distal 13q deletion resulting from the r(13) formation.

## Abbreviations

aCGH: array CGH; CGH: comparative genome hybridization; CNV: copy number variant; MAPD: median absolute pair-wise difference; SNP: single-nucleotide polymorphism.

## Competing interests

The authors declare that they have no competing interests.

## Consent

Written informed consent was obtained from the parents of our patient for publication of this case report and any accompanying images. A copy of the written consent is available for review by the Editor-in-Chief of this journal.

## Authors' contributions

CL consulted and interpreted the patient data of the chromosomal disease. FF performed the cytogenetic analysis and was a major contributor to the manuscript. LZ assisted in the aCGH test and data analysis. All authors read and approved the final manuscript.
